# Cause Mechanism of Metro Collapse Accident Based on Risk Coupling

**DOI:** 10.3390/ijerph19042102

**Published:** 2022-02-13

**Authors:** Ming Fang, Yi Zhang, Mengjue Zhu, Shaopei Chen

**Affiliations:** 1 School of Public Administration, Guangdong University of Finance & Economics, Guangzhou 510320, China; fangming@gdufe.edu.cn (M.F.); 20141258@gdufe.edu.cn (M.Z.); shaopei.chen@gdufe.edu.cn (S.C.); 2 Institute of Survey and Design, Guangdong Architectural Design & Research Institute Co., Ltd., Guangzhou 510010, China

**Keywords:** construction safety accident, analysis of the accident causes, 24Model, fault tree analysis, risk coupling

## Abstract

A metro collapse accident is the main type of metro construction accidents. How to scientifically analyze the key cause factors and their interaction coupling mechanism of the existing metro collapse accidents is crucial to reduce the occurrence of metro collapse. Based on the Fault Tree Analysis (FTA) and the Behavior security “2-4” Model (24Model), the FTA-24Model accident cause analysis framework was constructed by combing their respective characteristics. To be more specific, a logical analysis program was developed to analyze the accident causes by the four-module analysis method. An empirical study was carried out by taking the “12.1” major cave-in accident at the construction site of the Metro Line 11 in Guangzhou as an example. Compared with the case accident report, the FTA-24Model framework analysis method can not only systematically deduce the logical relationship between the accident causes and provides a panorama of the accident cause chain and its evolution process, but also identify the key causes of accidents and their coupling risk effects. For a metro construction accident, this method can not only effectively investigate the accident causes, but also provide a reference for the formulation of prevention strategies.

## 1. Introduction

At present, due to the fact that the period of metro construction for Britain, France, Germany, the United States, Spain, Japan, and other developed countries has passed, metro accidents in these countries mostly occur in the operation stage. Based on the analysis of 23 international typical major metro accidents in the past 30 years, the main reasons for the risk of major subway accidents are: artificial mechanical failure (36%); improper staff operation (30%); social disasters, such as fire, terrorist attacks (30%); and crowded factors (4%) [1]. In contrast, metro accidents in China often occur in the construction stage, because urban rail transit has been vigorously constructed and developed, under the background of deepening urbanization in China. As of 31 December 2020, in addition to Hong Kong, Macao, and Taiwan, a total of 44 cities in China had urban rail transit lines in operation.

The impact of metro construction on an urban environment cannot be ignored. Due to the deepening of urbanization in China and the complexity and particularity of metro engineering itself, such as long construction period, variable geological environment, and many risk factors [2], metro construction accidents have occurred frequently in recent years. According to incomplete statistics, there were 192 metro construction accidents in China from 2010 to 2019. Through the analysis of metro accident cases, it is found that the collapses are the most important accident type in metro engineering, accounting for 43% of accidents [3], which is a difficult problem to overcome in the current management of safety during metro construction. Therefore, China was selected as the main target region to carry out the analysis on the causes of metro construction collapse accidents.

Currently, quite a few scholars have conducted in-depth research on the causes of metro collapse accidents, based on fuzzy theory, probabilistic risk assessment, accident-causing theory, and other theories and methods.

To begin with, fuzzy theory-based risk assessment is an effective analysis method to assess and manage risk in metro construction projects. However, the existing fuzzy risk assessment studies on metro collapse accidents [4,5] have not been able to effectively process the data acquired from expert investigation method, for which the corresponding reliability is often difficult to guarantee.

Second, different methods of risk assessment have been developed on the basis of the probabilistic risk assessment, such as fault tree analysis (FTA), event tree analysis, risk matrix analysis, Monte Carlo methods, and Bayesian Networks. These methods have played a vital role in metro risk management [6,7,8,9,10]. However, for using probabilistic approaches of analysis, the reliability of the results is highly dependent on the validity and accuracy of the risk data used. In actual metro projects, owing to high levels of risk uncertainty, statistics data that fit the probabilistic modelling are difficult to acquire. Nevertheless, the qualitative method in FTA shows certain advantages in deducing and describing the logical relation of the causes of an accident. The top event of the fault tree denotes the most severe consequence of the accident. The second layer of the fault tree associated with each cause event represents subcause-events; their failure will cause a top event failure. Therefore, some scholars have combined FTA with fuzzy theory-based assessment and Bayesian Networks to evaluate the probability of collapse in metro construction [11].

Third, according to the accident-causing theory proposed after statistical analysis of the causes behind 75,000 injury accidents, approximately 80% of construction accidents are caused by human unsafe action (UA) and the remaining 20% are caused by the unsafe condition (UC) of objects [12]. This is summarized as the ‘8-to-2 principle’. Therefore, to avoid accidents, we must address the issues with human actions through the development of proper behavioral safety management policies and solve the problems with objects by engineering technologies. In this context, the term ‘human’ refers not only to an individual’s behavior but also to organizational behavior; ‘objects’ involve both environmental and material factors [13]. An in-depth analysis of recent engineering studies has found that complex geological environments, incorrect survey data, and unreasonable design schemes are the main risk factors for collapse in metro construction [14,15,16]. Among them, the geological environment is classified as a UC, while the other two are classified as UAs. This agrees with the ‘8-to-2 principle’. Therefore, accident-causing theory, and models such as the HFACS model [17], STAMP model [18,19] and 24Model of behavioral safety [20,21], have effectively analyzed various reasons attributable to humans and objects in metro accidents.

Nonetheless, accidents are, by nature, the result of multiple inter-coupling risk factors [22], which find specific expression in complex interaction paths between the factors. This coupling effect of risk is random, unexpected, irreversible, and systematic [23]. Therefore, the systematic method is comprehensive and realistic to explain the cause mechanism of accidents [24,25,26,27]. Among several available accident causation theories and models, the 24Model has the systematic characteristics of integrity, relevance, hierarchy and dynamism [28] for its two modes—static (linear) and dynamic (non-linear) [29]. In 24Model, the causes of the accident are attributed to four stages (immediate cause, indirect cause, radical cause, and root cause) at two levels (individual level and organizational level), which is the origin of the name 24Model. Therefore, the 24Model offers greater advantages than other accident causation theories and models in metro collapse accident, which have highly uncertain risk factors.

According to a previous study based on the 24Model regarding the causes of safety accidents in metro construction [20,21], UC are mainly caused by the UA or from habitual behavior. However, these studies did not consider the impact of the UC on the UA and were, therefore, limited in analyzing the inter-coupling effects between them. To combat this deficiency, FTA may be used. Through top–down deduction, the FTA determines causes (including UA and UC) and their interaction paths, stage by stage. Therefore, FTA is suitable to analyze the direct causes of an accident (UC and UA) and their coupling effects.

In view of the above by FTA and behavior security 24Model advantages and complementarities, some scholars have combined them and applied them to the cause analysis of chemical accidents [30], which could fully show the chain of accident cause and its evolution process. In this paper, the FTA method and 24Model were combined to construct a new accident cause analysis framework of FTA-24Model and clarify the cause analysis method and procedure of the metro collapse accidents. First, the section ‘Materials and Methods’ of this paper establishes the analysis framework and gives the specific four-module of the analysis framework. Then, the validity of the analytical framework was demonstrated by the case study. Second, the section ‘Results’ shows the advantages of FTA-24Model framework in analyzing the key causes of accidents and their coupling risk effects by comparing with the case accident reports. Third, the section ‘Discussion’ discusses the research results from three aspects: the causes of subway collapse, the improvement of 24Model and the scope of application of the FTA-24Model analysis framework. Finally, the section ‘Conclusion’ gives the main conclusions, limitations, and future directions of this paper.

## 2. Materials and Methods 

### 2.1. The Framework of Accident Cause Analysis

**Figure 1:**
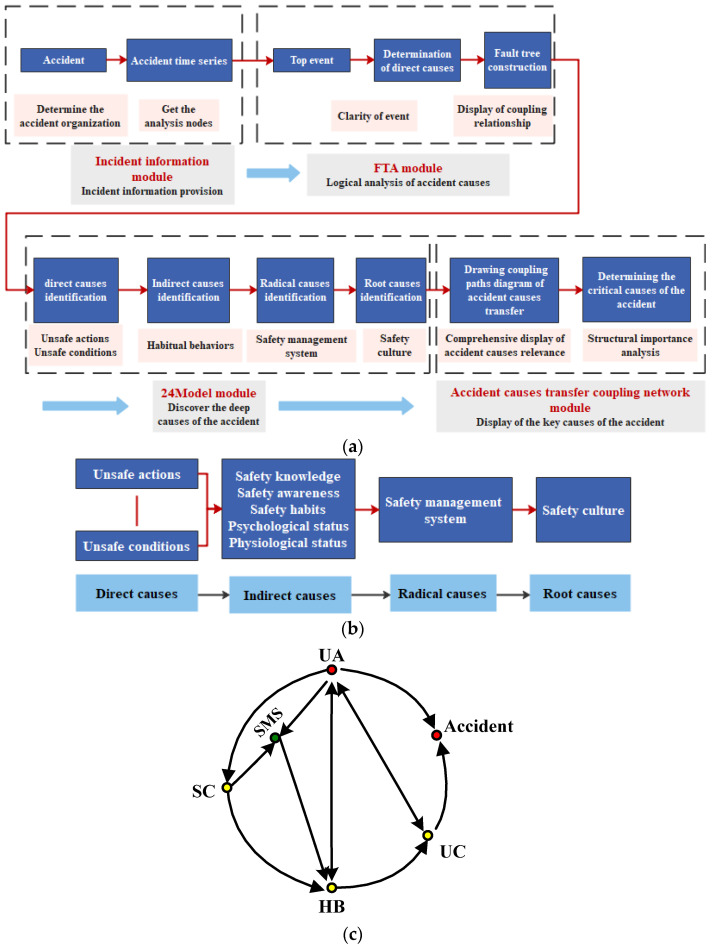
Model of the framework of accident cause analysis based on the FTA-24Model. (**a**) Framework analysis path diagram; (**b**) Static structure of 24Model module; (**c**) Dynamic structure of 24Model module.

The framework of accident cause analysis based on the FTA-24Model can be divided into four modules, including the accident information module, which can extract detailed information about the accident and its loss; the FTA module, which displays the logical relationship between the direct causes by drawing the fault tree; the 24Model module, which can explore the in-depth causes of accidents, including the safety management system and safety culture; and the accident causes transfer coupling network module, which presents the coupling path diagram of the accident causes to show the key causes of the accident (See Figure 1a). Among them, the 24Model module contains both static and dynamic structures, as shown in Figure 1b,c.

### 2.2. Accident Cause Analysis Process

#### 2.2.1. Accident Information Module

The accident report, the basis for accident cause analysis, can provide the basic information for the cause analysis in the subsequent modules. Therefore, the detailed level of an accident report directly determines the breadth and depth of accident cause analysis. The accident report module can be divided into two parts:

(i) Obtaining the basic information about an accident, including the basic situation of the accident, the accident process, the emergency response, and accident cause analysis. In this step, the accident organization and the direct cause of the accident can be clarified.
(ii) Drawing the accident time series diagram: According to the accident report, the typical events that contribute to the accident process are described in chronological order.

#### 2.2.2. The FTA Module

The FTA module, including the coupling paths analysis of direct accident causes and the fault tree establishment. This module is composed of the following three parts:

(i) Determination of top events: The object to be analyzed in this part is the top event. Through a comprehensive analysis of the accident, the most severe consequence of the accident is determined as the top event, which varies with the choice of time point. Therefore, the accident node can be determined with the accident time series diagram, and the top event can be selected.
(ii) Determination of cause events: The scattered information extracted from the accident report is integrated into cause events. The definition of the cause event should correspond to that in the 24Model, so as to facilitate further discussions about the in-depth causes. In general, cause events are classified into intermediate events and basic events. The former is both the cause of the top event and the result of the basic events, while the latter is the initial cause of the accident. Both are direct causes. For example, in the process of metro construction, grouting machine failure (the basic event) leads to the failure of timely support (the intermediate event), and the failure of timely support leads to the occurrence of seepage (the intermediate event), and finally leads to the occurrence of cave-ins (the top event). The basic events involve four aspects: mechanical (electrical) equipment failure or damage, human performance failure (operation, management, and command), poor environment, and poor quality of construction materials, which correspond to unsafe actions and unsafe conditions in the 24model [15] and are classified as internal organization causes [13].
(iii) Construction of the fault tree: The construction of the fault tree follows a certain logical relationship. By means of logical deduction, the top event, intermediate event, and basic event are scientifically and reasonably integrated into a logical diagram of the system [30]. The risk coupling theory is introduced here, and metro construction risks are divided into single-factor coupling risk, double-factor coupling risk and multi-factor coupling risk [2]. Single-factor coupling risk refers to the internal interaction of a single risk factor, such as human factor coupling risk (UA–UA) and environmental factor coupling risk (UC–UC). Double-factor coupling risk refers to the mutual influence of two risk factors, such as strong interaction between supporting measures and geological environment risk (UA–UC). Multi-factor coupling risk refers to the interaction between three or more risk factors, such as the interaction between safe construction management and control measures, construction according to standard specifications, and monitoring and early warning measures (UA–UA–UA).

#### 2.2.3. The 24Model Module

The purpose of this module is to identify the in-depth causes of the accident, so as to form a complete chain of accident causes. The direct causes (UA and UC) of accidents are identified by referring to the cause events in the fault tree, and then the indirect causes (habitual behaviors) and organizational causes (safety management and safety culture) of accidents are found based on the logical relationship in the 24Model. The analysis method can be used by the Why–Because method [30].

#### 2.2.4. The Accident Causes Transfer Coupling Network Module

This module aims to find the key causes of accidents and display the association between accident causes. The main content of this module is the transfer paths diagram of accident causes. In order to clarify the multiple paths of accident evolution and analyze the structural importance, the network analysis method is used to draw the associated path diagram of accident causes.

Through the analysis of the causes and prevention of metro construction accidents by using the FTA-24Model framework analysis method, various specific factors and their coupling paths, which cause the consequences of the accident, can be found out. In the prevention process of the accident, the success of accident prevention can be achieved to varying degrees by timely adjusting measures, changing relevant actions, or improving relevant conditions.

### 2.3. Empirical Research

This paper took the “12.1” major cave-in accident at the construction site of the Metro Line 11 in Guangzhou as an example for empirical research. On 1 December 2019, the cave-in accident of the cross passage of Shahe Station at the construction site of the Metro Line 11 in Tianhe District of Guangzhou was a major accident caused by complex geological conditions underground. The accident occurred in the city center of Guangzhou, and above the construction site was a main road. The accident caused the intersection of Guangzhou Avenue North and Yudong West Road to collapse. The accident, which caused a cleaning truck and an electric bike to fall into the pit, killed three people, and caused a direct economic loss of about CNY 20.047 million. The accident was a typical urban metro construction collapse accident, so it has a certain representativeness.

#### 2.3.1. Accident Information Module Analysis

Step 1: Obtaining the basic information about the accident case. According to the accident investigation report [31], the basic information about the “12.1” cave-in accident was obtained, as shown in Table 1.

**Figure 2:**
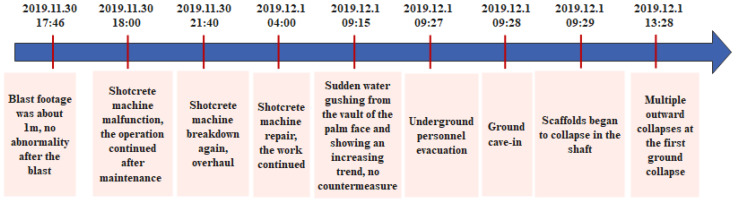
Accident time series diagram.

**Figure 3:**
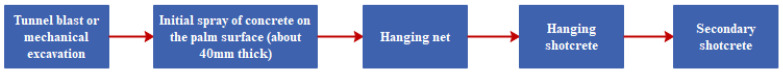
Construction process.

**Figure 4:**
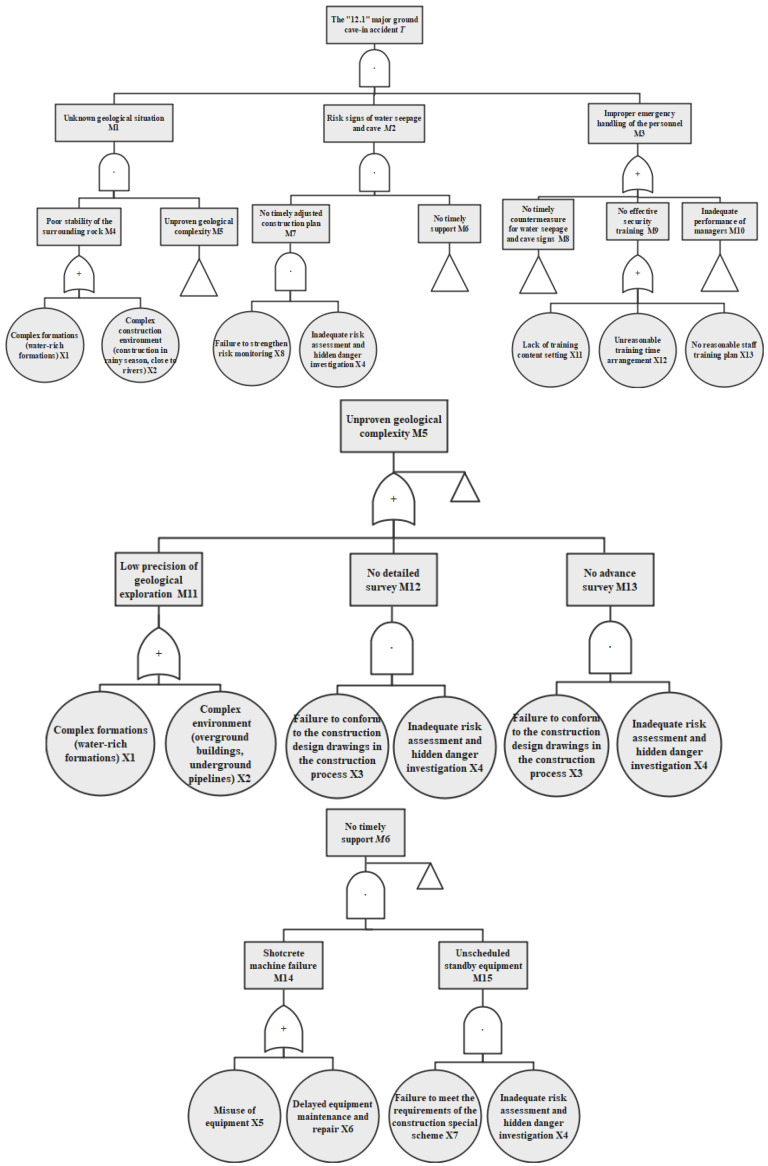

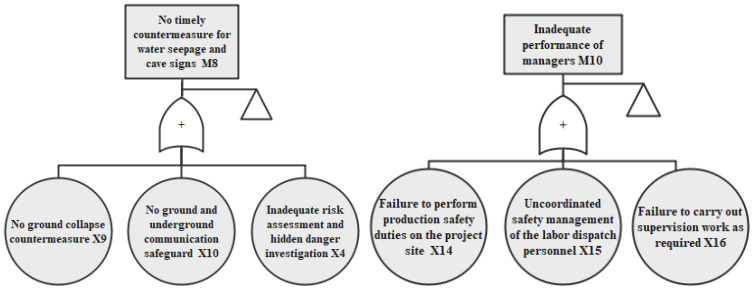
Fault tree diagram of the “12.1” major cave-in accident.

Step 2: Drawing the accident time series diagram. According to the accident report, the typical events that contribute to the accident process were described in chronological order, as shown in Figure 2. The main construction procedures were also extracted from the accident investigation report, as shown in Figure 3. Defining the accident time series and the main construction procedures would help to establish the basic events, intermediate events, and the top event in the FTA module. For example, according to the construction process in Figure 3, hanging shotcrete should be carried out in time after the completion of tunnel blasting and the initial spray of concrete on the palm surface. However, the time series of the accident in Figure 2 showed that the shotcrete machine failed twice (18:00 and 21:40 on 30 November) during the hanging concrete operation, so the shotcrete machine failure could be identified as an intermediate event in the FTA module (see M14 in Figure 4). Timely and effective maintenance was not carried out after the first failure of the shotcrete machine, which led to the occurrence of the second failure, and the maintenance of the shotcrete machine was not completed until 04:00 on 1 December. Therefore, the delayed equipment maintenance could be regarded as the basic event in the FTA module (see X6 in Figure 4). The most serious consequence in the time series of the whole accident process is ground cave-in (09:28, 1 December), which could be considered as the top event in the FTA module (see T in Figure 4).

#### 2.3.2. FTA Module Analysis

Step 3: Logical analysis of accident causes and drawing the fault tree. The sudden collapse at the intersection of Guangzhou Avenue North and Yudong West Road was determined as the top event. Through logical deduction, the scattered information extracted from the accident report was integrated into the cause events, and the fault tree of the collapse accident was drawn, as shown in Figure 4.

Through the analysis, 15 intermediate events and 16 basic events were identified. Among them, 25 events belong to Unsafe Acts (UA), including X3, X4, X5, X6, X7, X8, X9, X10, X11, X12, X13, X14, X15, X16, M3, M5, M6, M7, M8, M9, M10, M11, M12, M13, and M15, and 6 events belong to Unsafe Conditions (UC), including X1, X2, M1, M2, M4, and M14. The unsafe actions and unsafe conditions summarized in the above analysis may occur in all personnel, equipment, facilities, and environments of the organization along with the development of events. The logical relationship can be concluded as follows: unsafe actions lead to unsafe actions (UA–UA) for 24 items, unsafe actions lead to unsafe conditions (UA–UC) for 5 items, unsafe conditions lead to unsafe conditions (UC–UC) for 3 items, and unsafe conditions lead to unsafe actions (UC–UA) for 2 items, as shown in Table 2.

#### 2.3.3. 24Model Module Analysis

**Figure 5:**
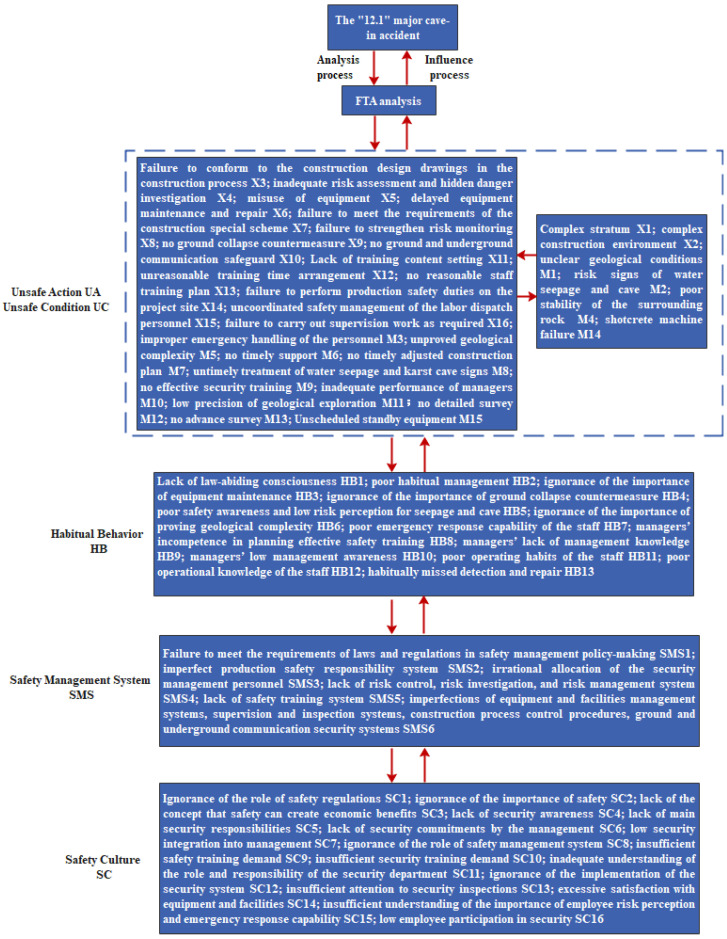
Cause analysis process and results of the “12.1” major cave-in accident based on the FTA-24Model.

Step 4: Identification of human factors and analysis of organizational causes. According to the accident cause items obtained by the FTA analysis and the logical relationship identified in the 24Model, the accident causal factors of each module were determined and coded. The specific analysis results are shown in Figure 5.

#### 2.3.4. Accident Causes Transfer Coupling Network Module Analysis

**Figure 6:**
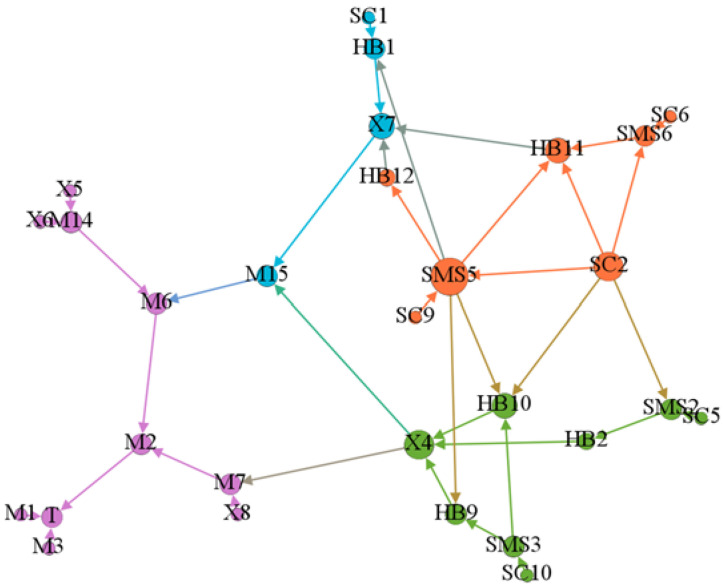
Results of the associated analysis of UC_M2_ accident causal factor.

Step 5: Identification of the correlation between accident causes. According to the logical relationship between the modules obtained from the analysis of the 24Model, the unsafe condition “UCM2” was taken as an example to further clarify the relationship between the accident causal factors through Gephi 0.9.2 software. Then, the directed graph with 29 nodes and 38 edges was drawn, as shown in Figure 6.

**Figure 7:**
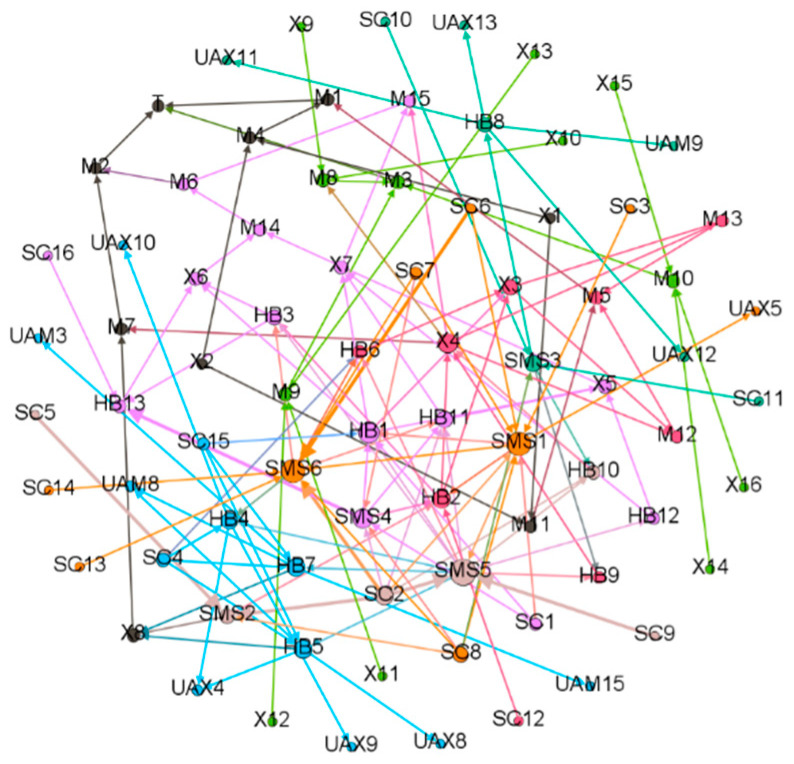
Association path diagram of accident causes.

According to the above analysis results, the transfer paths diagram of the accident causes was drawn. First, nodes (cause items) and directed edges (causal relationship) were input or imported, and the weight of edges was preliminarily determined as 1. The size of nodes depended on the size of the input and output degree. The maximum size of the node is set as 50 and the minimum size is set as 10. A total of 79 nodes and 134 directed edges were determined. Then, the Fruchterman Reingold dynamic modular layout was used to determine 7 modular units, and the analyzing degree was 1.0. The cluster distribution of the transfer paths for accident causes was obtained, as shown in Figure 7.

Figure 7 shows the key factors, including the direct factors at the personal level, such as a lack of risk assessment and hidden danger investigation (X4) and the failure to conform to the construction design drawings (X3); the indirect factors, such as a lack of law-abiding consciousness (HB1), poor habitual management (HB2); the factors of safety management at the organizational level, such as a lack of safety training system (SMS5) and the failure to meet the requirements of laws and regulations (SMS1) in safety management policy-making; and the factors of safety culture at the organizational level, such as the ignorance about the importance of safety (SC2) and the role of the safety management system (SC8). These factors had a significant correlation with other factors and had a great impact on the evolution path of the accident.

Step 6: Identify the key causes by structural importance calculation

**Figure 8:**
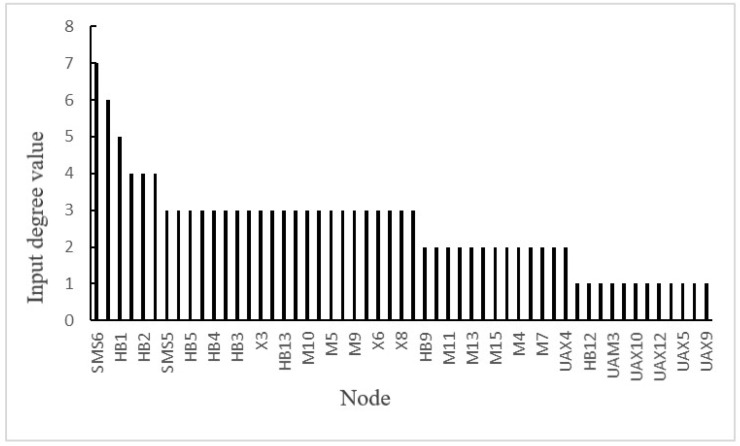
Input degree values of each node in causation network of accident.

**Figure 9:**
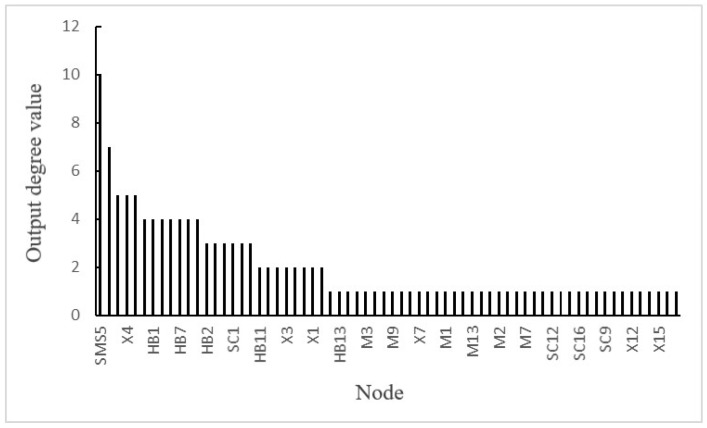
Output degree values of each node in causation network of accident.

(1) Calculation of the input degree value: In the accident causes model constructed in this paper, the input value represents the sum of all adjacent factor nodes that can transmit risks to this node [15]. According to the structure of Figure 7, the input degree value of each node could be calculated, as shown in Figure 8. The calculation results of the input degree value showed that the node SMS6 (imperfections of equipment and facility management systems, supervision and inspection systems, construction process control procedures, and ground and underground communication security systems) had the highest input degree value. Many factor nodes could transmit risks to the SMS6 node, such as SC2 (ignoring the importance of safety) and SC8 (ignoring the role of the safety management system). It was found that many factors could lead to the imperfection of equipment and facility management systems, supervision and inspection systems, construction process control procedures, and ground and underground communication guarantee systems, which, in turn, increased the possibility of accidents. From the perspective of risk coupling, the coupling relationship of the SMS6 node was multi-factor coupling risk, which was SC–SMS–SMS. In addition, the input degree values of SMS1 (safety management policy does not fully meet the requirements of laws and regulations) and HB1 (a lack of law-abiding consciousness) were relatively high. Among them, the coupling relationship of node SMS1 was double-factor coupling risk, which was SC–SMS; the coupling relationship of node HB1 was multi-factor coupling risk, which was SC–SMS–HB. Therefore, through the calculation of the input degree value, the factor nodes under the joint action of multiple risk factors can be highlighted to help decision-makers find the weak links in the management work and carry out corresponding risk control.

(2) Calculation of the output degree value: The output degree refers to a node’s ability to transmit risks to all adjacent factors [15]. The output degree value of each node in the network caused by the accident was calculated, as shown in Figure 9.

The results showed that the node with the highest output degree value was SMS5 (a lack of the security training system), followed by SC2 (the ignorance of the importance of safety) and X4 (inadequate risk assessment and hidden danger investigation). Among them, the coupling relationship of node SMS5 was double factor coupling risk, which was SMS–HB; the coupling relationship of node SC2 was multi-factor coupling risk, SC–SMS–HB; the coupling relationship of node X4 was the two-factor coupling risk, which was UA–UA. Therefore, these factor nodes demonstrated a strong initiative to transmit risks and were the main reasons for the occurrence of the accident.

**Figure 10:**
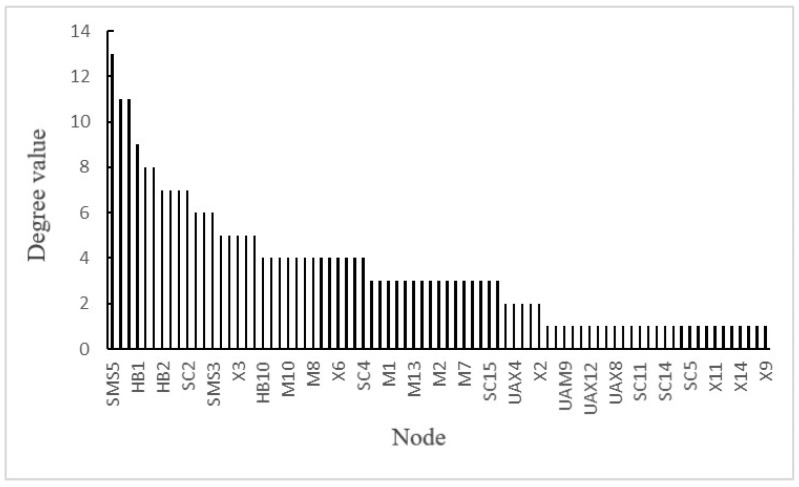
Degree values of each node in causation network of accident.

(3) Calculation of the degree value: The degree value of a node is the sum of the output degree value and input degree value of the node. The degree values of each node are shown in Figure 10.

Figure 10 shows that the degree values of nodes SMS5 (a lack of safety training system), SMS6 (imperfections of equipment and facility management systems, supervision and inspection systems, construction process control procedures, ground and underground communication security systems), and SMS1 (safety management policy does not fully meet the requirements of laws and regulations) are higher, suggesting that the imperfect safety management system was the main cause of the accident. To some extent, management factors affected human factors. For example, a lack of a safety training system indirectly affected the standardization of employees’ behaviors. Therefore, it is necessary to improve safety management, carry out effective safety training, and enhance the safety awareness of employees.

## 3. Results

In this paper, the causal factors of the accident analyzed by the FTA-24Model were compared with the description of the case accident report information, and the results are shown in Table 3.

It could be seen from Table 3 that the analysis of individual habitual behavior was ignored in the case accident report module, such as the precursor events that produce unsafe actions, namely the psychological and physical status, safety knowledge, ideological status, and so on. At the same time, the in-depth causes of the accident were ignored in the report, including a lack of organizational safety culture, which is not conducive to the control over the source of the accident. The identification of other causal factors in the case accident report module was also incomplete. The unsafe conditions without exact expression or the common unsafe actions conducted by other members of the organization were analyzed in the FTA-24Model, which made up for a lack of information in accident reports by clarifying the complex relationship between human factors and organizational factors. In addition, by using this framework of accident cause analysis, the coupling paths of accident causes could be displayed, the causal relationship between accident causes could be obtained, and then the key cause of the accident could be quantitatively analyzed through the calculation of structural importance.

## 4. Discussion

The four-step analysis process of the FTA-24Model framework was described in this paper. First, the basic information of the accident case was extracted through the accident report, and the time series diagram of the accident evolution was drawn. Second, the FTA deductive analysis process was used to identify the direct causes of the case, that is, UA, UC and their interactive coupling paths. Third, by using the 24Model, the causes at the organizational level were further explored. The human and organizational management factors in construction accidents were effectively identified, and the accident evolution process was scientifically described. Fourth, based on the above analysis results, the network analysis method was used to draw the accident causes transfer coupling network diagram, which could clearly show the multiple coupling paths of the accident cause, and find the key cause factors through the structural importance analysis.

### 4.1. Causes of Collapse Accident in Metro Construction

Through the analysis of the FTA-24Model framework method established in this paper, as shown in Table 2, the number of logical relationships UC–UA can be found is 2, which are X1–M11 and X2–M11, respectively. Among them, X1 refers to complex formations (water-rich formations), and X2 refers to complex construction environment (overground buildings, underground pipelines). Both causes lead to M11 (low precision of geological exploration). This is because the urban metro constructions are often located in the downtown area of the city, with risk factors such as busy above-ground traffic, numerous buildings, interlaced underground pipelines, and complex engineering geological and hydrological conditions. All of these risk factors are all classified as UC in the 24Model [13], and these risk factors may lead to low geological survey accuracy (UA), which may lead to unreasonable design scheme (UA). In this context, the construction of metro can easily lead to a series of environmental risks, such as ground collapse, cracking of surrounding buildings and so on. However, in the previous studies on the causes of various construction accidents based on the 24Model [15], more attention was paid to the analysis of UA–UC, such as mechanical equipment damage caused by improper human operation. Therefore, the existence of UC–UA is the characteristic of metro construction different from other construction accidents. For example, in the accident case of Section 2.3, as the ground above the subway construction site was the main road of the city (UC), it was impossible to carry out geological survey on the road and obtain accurate survey data (UA), so it was impossible to verify the existence of underground karst cave in this location.

Furthermore, according to the statistics of 247 metro construction accidents from 2003 to 2017 in China, the collapse accidents mainly occurred in March to May and November [32]. The main reason was that from March to May is the rainy season in South China and the middle and lower reaches of the Yangtze River, and the rainy weather in the middle and lower reaches of the Yangtze River lasts for a long time after November. Long-term rainfall makes the accident site soil loose (UC), which was easy to cause the ground collapse. The above conclusions indicate that the role of UC–UA is an important cause of metro construction collapse accident.

### 4.2. Improvement of the 24Model

**Figure 11:**
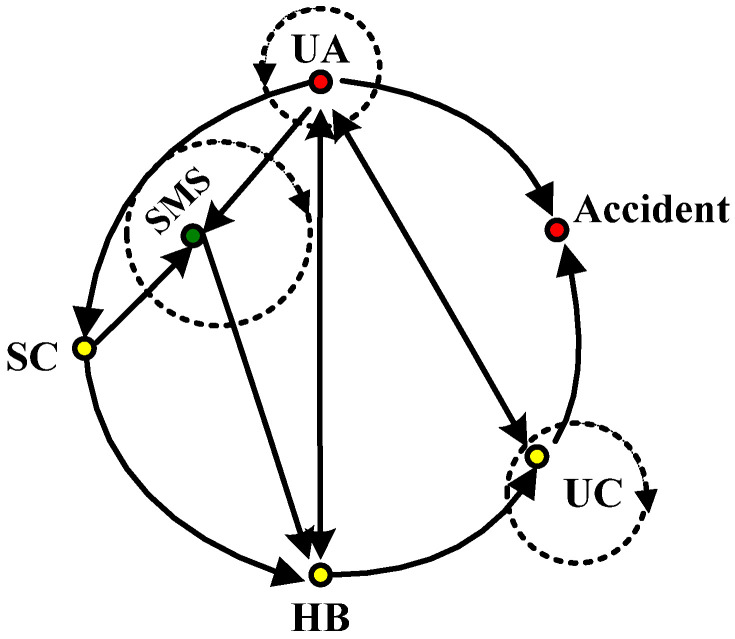
Improved 24Model module dynamic structure.

From the analysis of FTA module, it could be seen that for the direct causes of the metro collapse accident, there are not only the double-factor coupling relationship, such as UA–UC and UC–UA, but also the single-factor coupling relationship, UA–UA and UC–UC (see Table 2). Further, from the analysis of the accident causes transfer coupling network module, it could be seen that the coupling relationship of node SMS6 was a multi-factor coupling risk, which was SC–SMS–SMS. This multi-factor coupling relationship also contained the single-factor coupling relationship, SMS–SMS. Therefore, in the dynamic structure of the 24Model module (see Figure 1c), the action path of the above single-factor coupling relationship (see the virtual arrow in Figure 11) should be added, and the improved 24Model module dynamic structure is shown in Figure 11. The improved 24Model module can represent single-factor coupling risk, double-factor coupling risk and multi-factor coupling risk. Since the 24Model module is mainly derived from the 24Model, so the improvement of this module is equivalent to the improvement of the original dynamic form of the 24Model [29], which means that the 24Model not only has the systematic characteristics of integrity, correlation, hierarchy, and dynamism [28], but also has the characteristic of risk coupling.

### 4.3. Scope of Application of FTA-24 Model

Further comparative analysis found that the FTA-24Model accident cause analysis framework can be applied to various kinds of production safety accidents (including chemical accidents, coal mining accidents, construction safety accidents, etc.), because these accidents have similarities in the main disaster-causing factors, influencing mechanisms and consequences (as shown in Table 4). Increasing the use and promotion of the cause analysis framework of general production safety accidents will help scientific analysis and emergency decision-making of various events.

## 5. Conclusions

(1) Based on the FTA method and 24Model, this paper constructed the cause analysis framework of construction accidents, namely the FTA-24Model framework, which provided an effective way to explore accident causes and solve related security problems in the metro engineering. The FTA-24Model framework involved four modules, namely, the incident information module, the FTA logic module, the 24Model main structure module, and the accident causes transfer coupling network module, thus reasonably integrating scattered accident information, systematically displaying the logical relationship between the causes of the accident and the process of exploring the in-depth causes at the organizational level, and revealing the mechanism of the accident.
(2) By setting the six-step analysis method and conducting empirical research, 25 unsafe actions and 6 unsafe conditions were identified from 31 cause events. The 24Model module was used to analyze 13 indirect causal factors of personal habitual behaviors and 22 in-depth causal factors at the organizational level, and 134 correlation paths were involved in these causal factors. The analysis results of the correlation paths showed that the following causes of accidents had a greater impact on the accident process: inadequate risk assessment and hidden danger investigation (X4), a lack of awareness of compliance (HB1), poor habitual management (HB2), a lack of safety training system (SMS5), failure to meet the requirements of laws and regulations in safety management policy-making (SMS1), ignorance of the importance of safety (SC2), and ignorance of the role of the safety management system (SC8). According to the results of the correlation path analysis, multi-level accident prevention strategies could be formulated in the metro engineering.
(3) Compared with the case accident report, the FTA-24Model framework analysis method was more comprehensive and specific in identifying the causal factors of the accident and their coupling relationship, and could describe the accident process more accurately. However, in the application of the framework analysis method, it is necessary to pay attention to the delineation of the accident time node, and the definition of each event in the FTA method should be consistent with the concept of 24Model so that FTA and 24Model can be more effectively combined with each other.

Based on the above conclusions, using the FTA-24Model framework, through scientific analysis of the existing metro collapse accidents, valuable accident process information is obtained, and then the key cause factors and their interaction coupling mechanism are found, which has practical and guiding significance for improving the safety management effect of metro construction and reducing the occurrence of metro collapse accidents.

The Hain Rules believes that every accident has causes and signs, and that every accident can be avoided. A large number of practices have shown that basically all metro construction accidents have foreboding, which can be reflected in monitoring data and engineering phenomena [33,34]. Therefore, safety risks of metro construction can be monitored, and accidents can be avoided through risk analysis, evaluation, and management. On this basis, the accident database can be further established. Combined with the local reality, to identify similar cases and analyze the causes of accidents can provide reference for the future metro construction, in order to reduce the possibility of similar accidents. At the same time, it can provide experience for solving problems in other similar cases. In this paper, FTA-24Model framework was used to analyze the collapse accident case of metro construction, which could provide experience for the prevention of collapse accident. However, this paper failed to analyze other accident types of metro construction, such as falling from height and object strike accidents. Therefore, it is necessary to further analyze other types of metro construction accidents with the FTA-24Model accident cause analysis framework, so as to broaden the application of this accident cause analysis framework. In addition, the comparative study on the causes of metro construction accidents in China and other countries will be carried out in the future.

## Figures and Tables

**Table 1:** Basic information about the accident case.

Accident Organisation	Accident Occurrence Time	Accident Type	Accident Level	Accident Consequences	Accident Nature
China Railway Group Limited	1 December 2019	Collapse	Major accident	3 deaths, direct economic loss of about CNY 20.047 million	Production safety accident

**Table 2:** Fault tree analysis results.

Statistical Item	Category	Quantity	Specific Event
Cause items	Intermediate events	15	
	Basic events	16	
Direct causes	UA	25	X_3_, X_4_, X_5_, X_6_, X_7_, X_8_, X_9_, X_10_, X_11_, X_12_, X_13_, X_14_, X_15_, X_16_, M_3_, M_5_, M_6_, M_7_, M_8_, M_9_, M_10_, M_11_, M_12_, M_13_, M_15_
	UC	6	X_1_, X_2_, M_1_, M_2_, M_4_, M_14_
Logical relationship	UA–UA	24	As shown in Figure 4
	UA–UC	5	
	UC–UC	3	
	UC–UA	2	

**Table 3:** Comparison of the accident cause analysis results obtained by different analysis methods.

Accident Causes	Information Obtained Based on Incident Reports	Based on the FTA-24Model	Comparison of the Results (the Missing Items of Accident Report Information)
Unsafe action	① Low precision of geological exploration M11; ② no detailed survey M12; ③ no advance survey M13; ④ unscheduled standby equipment M15; ⑤ no ground collapse countermeasure X9; ⑥ no ground and underground communication safeguard X10; ⑦ failure to perform production safety duties on the project site X14; ⑧ uncoordinated safety management of the labor dispatch personnel X15; ⑨ failure to carry out supervision work as required X16	25 items (as shown in Figure 5)	Hidden unsafe actions: mistakes in equipment use, maintenance and operation, and training plan formulation
Unsafe condition	① Complex formation X1; ② complex construction environment X2; ③ unknown geological situation M1; ④ risk signs of water seepage and cave M2; ⑤ poor stability of the surrounding rock M4; ⑥ shotcrete machine failure M14	6 items (as shown in Figure 5)	—
Personal habitual behavior	—	13 items (as shown in Figure 5)	Unsafe action premise: knowledge, consciousness, habits, physical, psychological, and other factors
Safety management system	① Failure to meet the requirements of laws and regulations in safety management policy-making SMS1; ② imperfect production safety responsibility system SMS2; ③ irrational allocation of the security management personnel SMS3; ④ lack of the risk control and the risk investigation and management system SMS4; ⑤ lack of the safety training system SMS5	6 items (as shown in Figure 5)	Equipment and facility management systems, supervision and inspection systems, construction process control procedures, and ground and underground communication security systems were not perfect
Safety culture	—	16 items (as shown in Figure 5)	Integration of safety into safety management, safety commitments, and main security responsibilities

**Table 4:** Main parameters of various production safety accidents.

Item	Production Safety Accident
Main elements involved	Man, machine, material, method, medium, etc.
Causal factors	The abnormal action or condition of each element
Duration time	Generally shorter
Prevention time	Generally production time
Influence scope	Generally small
Consequences	Casualties and property losses

## Data Availability

We declare that all data, models, and code generated or used during the study appear in the submitted article.
